# Vitamin E Inhibits Oxidative Stress and Inflammation in Stress‐Induced Gastritis via Modulating Nrf2 and NF‐κB Signalling Pathways

**DOI:** 10.1111/jcmm.70463

**Published:** 2025-05-26

**Authors:** Xiaolin Xie, Si Zhao, Rui Fang, Huan Chen, Han Zhang, Xue Wang, Jun Gao, Yan Liu, Zihao Cai, Ming Zhang, Bing Xu, Yuzheng Zhuge

**Affiliations:** ^1^ Department of Gastroenterology, Nanjing Drum Tower Hospital, Clinical College Jiangsu University Nanjing Jiangsu China; ^2^ Department of Gastroenterology, Nanjing Drum Tower Hospital, Affiliated Hospital of Medical School Nanjing University Nanjing Jiangsu China; ^3^ Department of Gastroenterology, Nanjing Drum Tower Hospital, Clinical College Nanjing Medical University Nanjing Jiangsu China

**Keywords:** NF‐κB, Nrf2, stress‐induced gastritis, vitamin E

## Abstract

The incidence of stress‐induced gastritis is gradually increasing. Vitamin E (VE) is widely used in inflammatory diseases due to its efficient antioxidant and anti‐inflammatory effects. Here, we investigated the protective role of VE on stress‐induced gastritis and its potential mechanisms. Mice were subjected to high‐intensity stress caused by the forced swim test (FST) and gavaged with VE (300 mg/kg) at different time points. The results showed that VE significantly alleviated stress‐induced gastric mucosal injury and related histopathological changes. Additionally, the expression levels of nuclear factor erythroid 2‐related factor 2 (Nrf2), haem oxygenase‐1 (Hmox1) and NAD(P)H:quinone oxidoreductase 1 (NQO1) were upregulated in the administrated groups, while the nuclear factor kappa B (NF‐κB) signalling pathway was inhibited, manifested as the expression level of p‐NF‐κB p65 protein decreased. Furthermore, VE reduced the infiltration of macrophages in gastric tissue, followed by a synchronous decrease in the expression level of interleukin‐1 beta (IL‐1β) protein. Importantly, the detection of cell death by TUNEL assay and SYTOX green staining demonstrated that VE reduced cell death of gastric tissue and subsequently downregulated the pro‐apoptotic factor BCL2‐associated X Protein (Bax). Hence, our study suggested that VE has an outstanding preventive and therapeutic effect on stress‐induced gastritis via promoting Nrf2 and inhibiting NF‐κB signalling.

**Abbreviations**
BAX: Bax pro‐apoptotic factor BCL2 associated X Protein
DAB: diaminobenzidine
ddH_2_O: deionised distilled water
dUTP: deoxyuridine triphosphate
H_2_O_2_
: hydrogen peroxide
Hmox1: haem oxygenase‐1
H&E: haematoxylin and eosin
IL‐1β: interleukin‐1 beta
NF‐κB: nuclear factor kappa B
NQO1: NAD(P)H:quinone oxidoreductase 1
Nrf2: nuclear factor erythroid 2‐related factor 2
OCT: optimum cutting temperature
OD: optical density
PBS: phosphate‐buffered saline
PVDF: polyvinylidene difluoride
qRT‐PCR: quantitative Real‐time PCR
ROS: reactive oxygen species
SGML: stress‐induced gastric mucosal lesion
TDT: terminal deoxynucleotidyl transferase
TNF‐α: tumour necrosis factor‐alpha
VE: vitamin E
WIRS: water immersion restraint stress

## 1 Introduction

Stress‐induced gastritis, a subtype of gastritis in digestive system diseases, is an inflammatory response to the gastric mucosa damage caused by multiple stresses [1]. However, the pathogenesis of stress‐induced gastritis has not been completely elucidated.

It is well established that oxidative stress plays a pivotal role in the development of stress‐induced gastritis. Reactive oxygen species (ROS) can damage the gastric mucosa by the combined mechanism of enhancing lipid peroxidation and weakening mucosal antioxidant defences [2, 3]. The nuclear factor erythroid 2‐related factor 2 (Nrf2) signalling pathway, as a significant regulator of the body's response to oxidative stress, can activate its downstream target genes, such as haem oxygenase‐1 (Hmox1) and NAD(P)H:quinone oxidoreductase 1 (NQO1), which help eliminate excess ROS and reduce oxidative damage [4, 5]. Thus, the Nrf2/Hmox1/NQO1 signalling pathway is crucial in alleviating oxidative stress and protecting the gastric mucosa.

In addition, inflammation is also believed to be involved in the pathogenesis of stress‐induced gastritis [6]. It is generally known that the nuclear factor kappa B (NF‐κB) is a transcription factor that plays an important regulatory role in inflammatory responses [7, 8, 9]. Numerous evidence has revealed that the activation of NF‐κB can induce the generation of a large number of pro‐inflammatory signalling molecules, such as tumour necrosis factor‐alpha (TNF‐α) and interleukin‐1 beta (IL‐1β), leading to an inflammatory response [10, 11]. Therefore, inhibiting the NF‐κB signalling pathway represents a promising strategy to reduce inflammation and protect the gastric mucosa from further damage.

Vitamin E (VE) is an effective and widely recognised lipid‐soluble vitamin with valuable antioxidant and anti‐inflammatory properties, which has been frequently reported on its protection in various inflammatory diseases [12, 13, 14, 15]. However, its mechanism of action in combating gastric tissue damage caused by stress remains to be studied. Based on the above, we hypothesise that VE potentially inhibits the oxidative stress and inflammation during the development of stress‐induced gastritis by regulating Nrf2/Hmox1/NQO1 and NF‐κB signalling pathways.

Currently, the risk of suffering from stress‐induced gastritis is gradually increasing. Among critically ill patients, there is an urgent need for effective therapeutic strategies [16, 17, 18]. Due to the complexity of the etiological mechanism and the diversity of epidemiology, the formulation of prevention and treatment of stress‐induced gastritis is still a great challenge. We mainly conduct research on the mechanism of VE in the prevention and treatment of stress‐induced gastritis, which is of great significance.

## 2 Materials and Methods

### 2.1 Experimental Animals

A total of 20 male C57BL/6 mice (aged 8 weeks, 20–25 g) free of specific pathogens were purchased from Nanjing Qingzilan Technology Co. Ltd. (Jiangsu, China). To adapt to the environment, mice were raised under a standard animal laboratory environment for 1 week with ad libitum access to food and water. All current experimental procedures were carried out in accordance with the ethical principles followed by the Research Ethics Committee of Nanjing Drum Tower Hospital.

### 2.2 Experimental Design

Prior to the experiment, all experimental mice were prohibited from eating but not from drinking for 12 h. The stress measure given to the mice in this experiment was to place mice in cold water close to 10°C for periodic swimming with a cycle of swimming for 0.5 h and resting for 0.5 h. Such a repeated cycle lasted for a total of 4 h. Twenty mice were completely randomly divided into four groups, with five mice in each group. Group A was the Control group, which did not take measures to give stress, drug pretreatment or treatment. Group B was the Stress group, given appropriate stress without drug pretreatment or treatment. Group C was assigned to the Stress + VE (Zhejiang Pharmaceutical Co. Ltd., China) group. Before applying a certain stress, mice were administered VE (300 mg/kg) dissolved in an appropriate amount of corn oil by gavage. Group D was a Stress + VE Treatment group. After 2 h of stress given, mice were gavaged with VE (300 mg/kg) dissolved in corn oil in moderation. After 4 h of the experiment, all mice were euthanised by the cervical dislocation method and followed by laparotomy to remove the fresh stomachs which was washed with phosphate‐buffered saline (PBS). After that, stomachs were cut along the side of lesser curvature to separate the gastric tissue. One portion of the gastric tissue sample was stored in a 10% formalin solution, while the second portion of that was placed immediately in liquid nitrogen for quick freezing and then stored at −80°C until further analysis. The third portion was processed fresh. The tissues were embedded in optimum cutting temperature (OCT) compound and cut into 5‐μm thick cryosections using a cryostat.

### 2.3 Macroscopic Examination

The tissue damage, including hyperaemia, mucosal haemorrhagic spot and ulcer formation, was observed visually in the intact and incised stomach samples taken from each group. Then, the effect of drug therapy was evaluated macroscopically.

### 2.4 H&E Staining and Histological Evaluation

In short, the gastric tissue samples of each group were fixed in 10% formalin and then embedded in paraffin to be sliced into sections with a thickness of 5 μm. These sections stained with haematoxylin and eosin (H&E) were observed under a light microscope for histopathological evaluation. The reference for microscopic scoring of gastric damage we are interested in is shown in Table 1. As mentioned earlier, the total score ranges from 0 to 14 points, with the scoring criteria based on following evaluation indicators in the 1 cm segment of each tissue slice. Haemorrhage span (0–4 points): If one‐fourth of the sample's horizontal span includes bleeding areas, one point will be awarded. A score of 4 indicates that more than three‐fourths of the span are occupied by bleeding. Oedema span (0–4 points): The standards of grading are similar to the haemorrhage span stated above. Epithelium condition (0–3 points): Grades 1–3 (mild to severe) are based on a review of a series of slices, taking into account the severity and diffuse nature of lesions. Inflammatory cell infiltration (0–3 points): When the mild increase of inflammatory cells exceeds the observer's perception of normal existence, it is rated as 1 point, and a score of 3 is given when there is a severe increase in the infiltration and even extends into the subjacent glands. The final score is the sum of the individual scores from these categories, with 0 representing no abnormalities and 14 indicating severe damage [19, 20].

**TABLE 1:** The outline of histology assessment.

Evaluation index	Scores
Haemorrhage span	0–4
Oedema span	0–4
Epithelium	0–3
Inflammatory cells	0–3

### 2.5 RNA Extraction and qRT‐PCR

According to the manufacturer's protocol, total RNA from gastric tissue was extracted separately by TRIzol reagent (TaKaRa, Kusatsu, Japan). Using SYBR Green PCR Master‐Mix, quantitative real‐time PCR (qRT‐PCR) was performed on LightCycler 96 Fluorescent PCR. Melt curve analysis represented the specificity of the product. β‐actin was used as the reference gene to normalize the expression levels of target genes using the 2^−ΔΔCt^ method. The primers used in this study are listed in Table 2.

**TABLE 2:** Primer sequences for qRT‐PCR.

Gene	Forward (5′–3′)	Reverse (5′–3′)
Hmox1	CACGCATATACCCGCTACCT	CCAGAGTGTTCATTCGAGCA
NQO1	TTCTCTGGCCGATTCAGAGT	GGCTGCTTGGAGCAAAATAG
β‐actin	GCTACAGCTTCACCACCACAG	GGTCTTTACGGATGTCAACGTC

### 2.6 Western Blot Analysis

Briefly, total proteins were extracted from the remaining gastric tissue. According to the manufacturer's instructions, the tissue was first lysed. Then, the total proteins were extracted after centrifugation and the protein concentration was determined subsequently. After that, the samples were mixed with the appropriate volume of loading buffer. We used 10% SDS‐PAGE gel to isolate the proteins, followed by transferring them onto polyvinylidene difluoride (PVDF) membranes. After that, the PVDF membranes were blocked in 5% skim milk for 1–2 h. After a certain amount of washing, the proteins on the membrane were incubated with the corresponding primary antibody in a shaking bed at 4°C overnight and were incubated with the appropriate secondary antibodies at room temperature for 1–2 h the next day. The following primary antibodies were used:anti‐NF‐κB p65 (1:1000, CST, Cat#8242S), anti‐p‐NF‐κB p65 (1:1000, CST, Cat#3033S), anti‐IL‐1β (1:1000, Immunoway, YT5201), anti‐Bax (1:1000, CST, Cat#14796S) and anti‐β‐actin (1:3000, Proteintech, 81115‐1‐RR). β‐actin and p65 were used as a loading control. Eventually, the protein bands were developed, and Image J was used to analyse the optical density (OD) of the protein bands. Data are shown as target protein (OD)/β‐actin (OD) or p65 (OD).

### 2.7 Immunohistochemical Analysis

The paraffin‐embedded gastric tissue was cut into 5 μm sections. After baking at 60°C for 1 h, the sections were deparaffinised using xylene and a graded ethanol series, then rehydrated with PBS. Antigen retrieval was performed by heating the slides in citrate buffer to unmask the antigens. Non‐specific binding sites were blocked by incubating the sections with 5% BSA for 30 min. The primary antibodies were then applied and incubated overnight at 4°C. Following washing, the appropriate secondary antibodies were incubated at room temperature for 1 h. For visualisation, diaminobenzidine (DAB) was used, and the staining was observed under a microscope. Finally, the sections were dehydrated through a graded ethanol series, cleared with xylene and mounted with a mounting medium before being examined under a microscope [21]. The following primary antibodies were used: rabbit polyclonal anti‐F4/80 (1:1000, Servicebio, GB113373) and rabbit polyclonal anti‐Nrf2 (1:1000, Servicebio, GB113808).

### 2.8 TUNEL Assay

Following the instructions of the apoptosis detection kit (Servicebio technology CO. LTD, China), TUNEL assay of gastric tissue was performed. Briefly, paraffin sections of gastric tissue were dewaxed with xylene and incubated with protease K. Later, they were all equilibrated at room temperature after blocking endogenous peroxidase with 3% hydrogen peroxide (H_2_O_2_). The TUNEL reaction solution was prepared with terminal deoxynucleotidyl transferase (TDT), deoxyuridine triphosphate (dUTP) and buffer at a 1:5:50 ratio. Freshly prepared DAB chromogenic solution was used to develop the reaction signals, and nucleus was counterstained with haematoxylin staining solution for 1 min.

### 2.9 SYTOX Green Staining

SYTOX green (MKBIO, MX4228), a green nuclear acid staining solution that can easily pass through the damaged cell membranes but not live ones, is used as an indicator for dead cells in a cell population [22]. The sections of gastric tissue were restored at room temperature and washed three times with PBS for 5 min each time. SYTOX green stock solution was diluted with deionised distilled water (ddH_2_O) at a ratio of 1:5000. A sufficient staining solution covering the tissue was incubated at room temperature for 30 min without light. The tissue sections were then washed three times with PBS for 5 min each time. After that, nuclei were counterstained with DAPI (4',6‐diamidino‐2‐phenylindole), a DNA‐spercific fluorescent dye, for 5 min. Finally, they all were observed under a fluorescent microscope [49].

### 2.10 Statistical Analysis

All data were expressed as the mean ± SD. Data were analysed with one‐way ANOVA using Graphpad Prism (Graphpad Software Inc., California, USA). *p* values < 0.05 were considered statistically significant.

## 3 Results

### 3.1 Gastric Injury

**FIGURE 1:**
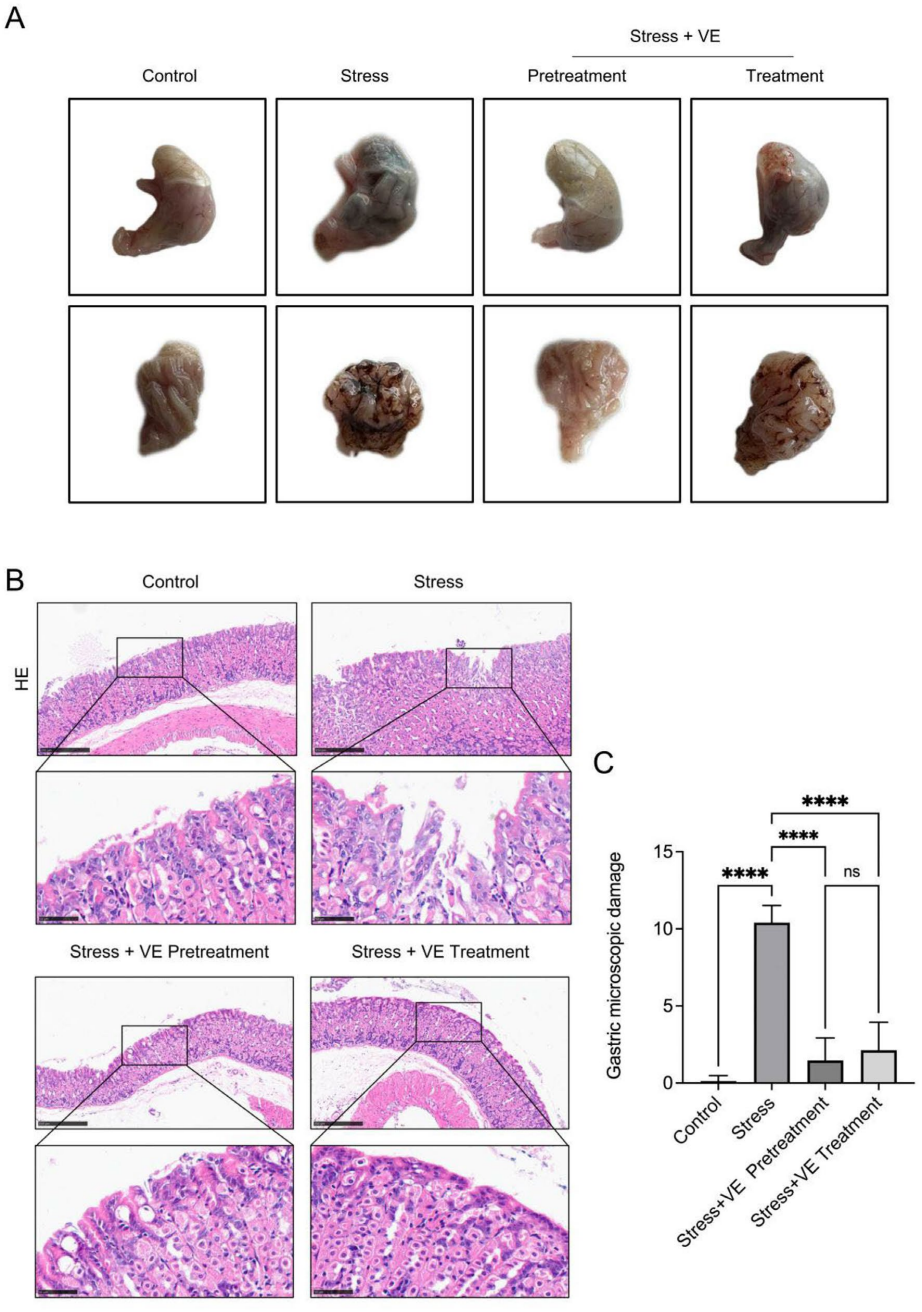
Effect of VE on the stress‐induced gastric gross, histopathology and microscale scoring. Representative photographs are shown. (A) Macroscopic examination of the stomach. (B) H&E staining of histopathological sections of gastric tissue. Magnification: ×10, scale bar =250 μm; magnification: ×40, scale bar = 50 μm. (C) Microscopic scoring of gastric damage. All data are expressed as the mean ± SD. ****p < 0.0001.

In accordance with expectation, subjected to repeated and sustained stress, the mice developed, as we observed from Figure 1A, a certain degree of significant haemorrhagic gastric injury. Mice that only received stress showed stomach swelling, obvious gastric mucosal bleeding, ulcer and other injuries. However, the mice treated with VE did not show the significant changes of macroscopic findings as mentioned above, but were close to normal gastric mucosa that reflected extremely mild haemorrhagic gastric injury.

### 3.2 Histological Findings and Microscopic Scoring of Gastric Damage

The gastric tissue sections were all stained by H&E so that we were able to evaluate the pathological morphology of the gastric tissue of mice. As Figure 1B represented, we concluded that the gastric sections in the control group showed intact mucosal tissue accompanied by well‐developed gastric glands. On the contrary, the pathological characteristics of gastric tissue damaged due to stress were manifested as the destruction of the mucosal structure, extensive bleeding and infiltration of inflammatory cells. Notably, the gastric histopathological analysis showed only mild inflammation in both groups of mice administered VE. In order to compare the degree of gastric tissue injury in each group, we performed a micro‐score on the obtained pathological results. The detailed grading criteria are provided in the Materials and Methods section. The results are quantified and presented as Figure 1C. As expected, the results indicated that the damage score of gastric tissue in mice treated with VE was significantly reduced relative to those only treated with stress (*p* < 0.0001), which confirmed that VE probably had a remarkable slowing or even inhibitory effect on the pathological development of stress‐induced gastritis. However, there was no significant difference between the pretreatment group and the treatment group (*p* > 0.05).

### 3.3 VE Inhibits Oxidative Stress in Stress‐Induced Gastritis via Promoting the Nrf2/Hmox1/NQO1 Signalling Pathway

**FIGURE 2:**
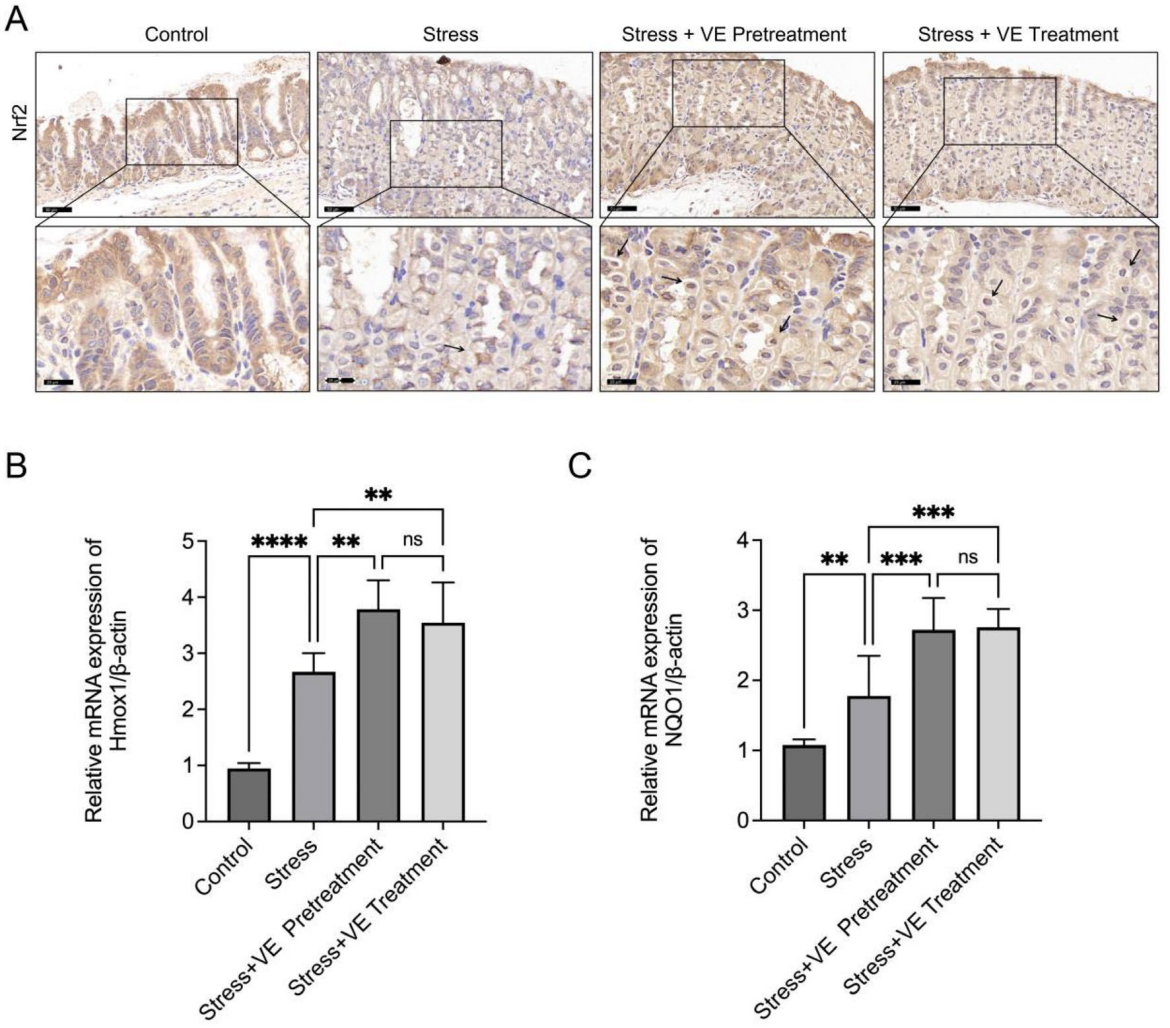
VE promotes Nrf2/Hmox1/NQO1 signalling pathway. (A) Representative immunohistochemical staining of Nrf2 in the gastric tissues. Arrowheads indicate the Nrf2‐positive cells in the nucleus. Scale bar = 50 μm; scale bar (partial enlarged view) = 25 μm. (B) Relative mRNA expression of Hmox1 and NQO1. All data are expressed as the mean ± SD. **p < 0.01, ***p < 0.001, ****p < 0.0001.

Considering the crucial role of Nrf2 in regulating antioxidant responses, its activation is central to alleviating oxidative stress and inflammation. To investigate the involvement of Nrf2 in the protective effects of VE against stress‐induced gastritis, the expression level of Nrf2 in gastric tissue of stress‐induced gastritis was analysed by immunohistochemistry. As observed in Figure 2A, there was a slight increase in the Nrf2 expression in the group subjected to stress compared to the control. Additionally, the result also showed a marked increase in the expression of Nrf2 in both groups treated with VE relative to the stress group. Together with the analysis of Nrf2, we measured the expression levels of downstream genes of it. The data illustrated in Figure 2B indicated that there was a significant upregulation of the expression of Hmox1 (*p* < 0.01) and NQO1 (*p* < 0.0001) mRNA levels in the simple stress group relative to the control one. What is more, testing in mice treated with VE revealed a further increase in the expression of Hmox1 (*p* < 0.001) and NQO1 (*p* < 0.01). However, no statistically significant differences in the expression levels of Hmox1 and NQO1 were observed between the two VE‐treated groups (*p* > 0.05). These results suggested that the inhibition of oxidative stress by VE is likely mediated through the promotion of the Nrf2/Hmox1/NQO1 signalling pathway.

### 3.4 VE Suppresses Gastric Inflammation Predominantly via Inhibiting the NF‐κB Signalling Pathway

**FIGURE 3:**
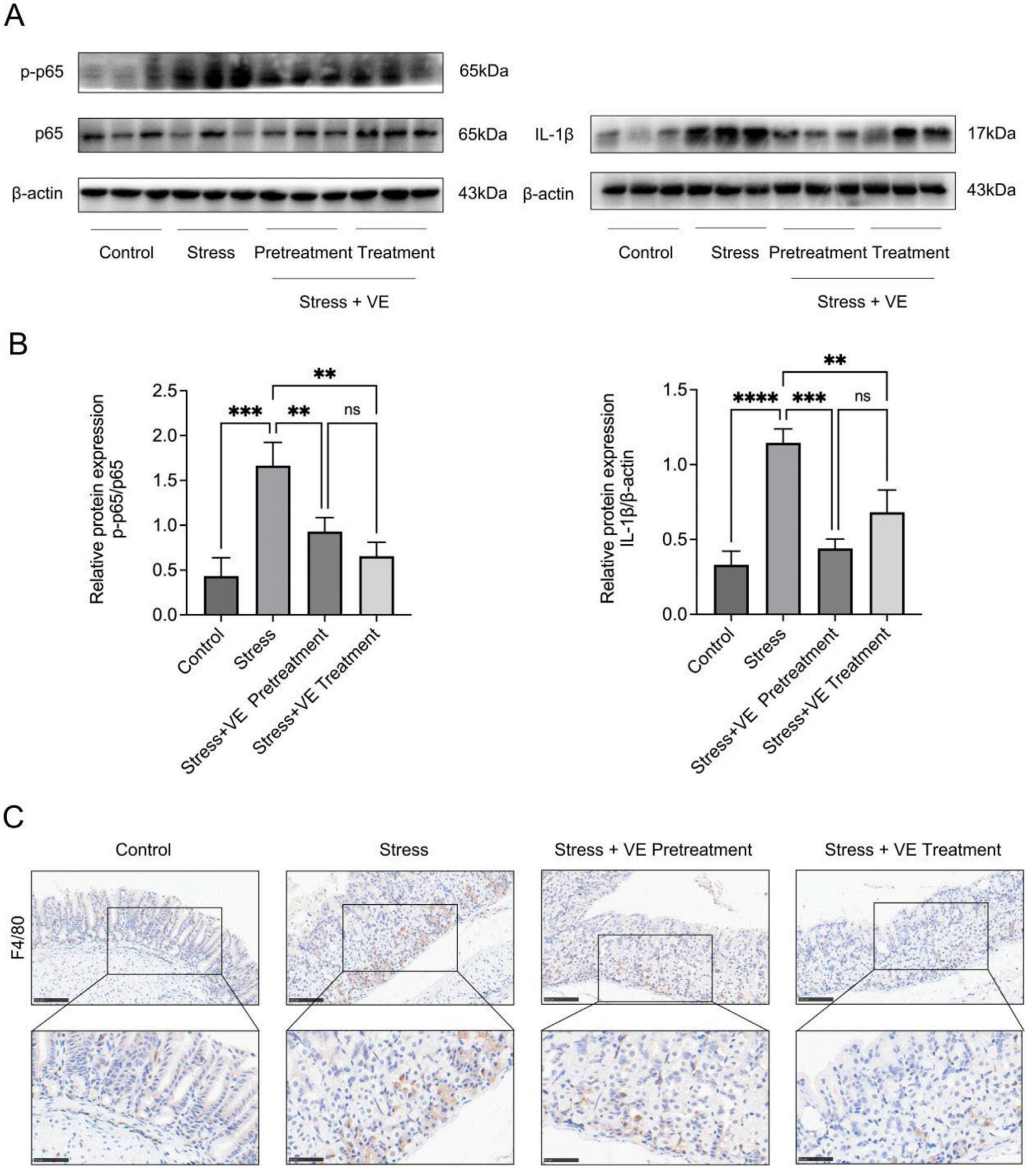
Effect of VE on the gastric protein expression of NF‐κB pathway‐related proteins and infiltration of macrophages in gastric tissue. (A) Western blot for NF‐κB pathway‐related proteins (NF‐κB p65, p‐NF‐κB p65 and IL‐1β). (B) The quantification of western blot for NF‐κB pathway‐related proteins (p‐NF‐κB p65 and IL‐1β). All data are expressed as the mean ± SD. **p < 0.01, ***p < 0.001, ****p < 0.0001. (C) Infiltration of macrophages in gastric tissue as determined by immunohistochemistry. Representative macrophages F4/80 staining photographs of gastric tissues in different groups were presented. Magnification: ×20, scale bar =100 μm; magnification: ×40, scale bar =50 μm.

Given the significance of NF‐κB in inflammation, we focused on assessing its activation in disease progression. Initially, we assessed the phosphorylation level of p65, a critical mediator of the pathway. The conclusion drawn from Figure 3A,B showed that the phosphorylation level of p65 in the stress group was increasing significantly (*p* < 0.001). Due to the intake of VE, NF‐κB signalling pathway was inhibited accordingly compared with the stress group. The phosphorylation level of p65 was significantly downregulated (*p* < 0.01). What's more, there was a substantial decrease in IL‐1β expression which is a downstream factor of the pathway, with a greater decline in the pretreatment group (*p* < 0.001) than in the treatment group (*p* < 0.01). Similarly, we were also unable to conclude that there were statistically significant differences in the phosphorylation level of p65 and the expression of IL‐1β between the pretreatment and treatment groups (*p* > 0.05). We delighted to explore whether VE has an impact on the activation of macrophages in stress‐induced gastritis. As we observed from Figure 3C, the results of the immunohistochemical analysis showed that the infiltration of macrophages in mice pretreated and treated with VE was significantly reduced compared to the mice under simple stress. These results indicated that VE had a certain inhibitory and protective effect on stress‐induced gastric inflammation.

### 3.5 VE Reduces Apoptosis in Stress‐Induced Gastritis

**FIGURE 4:**
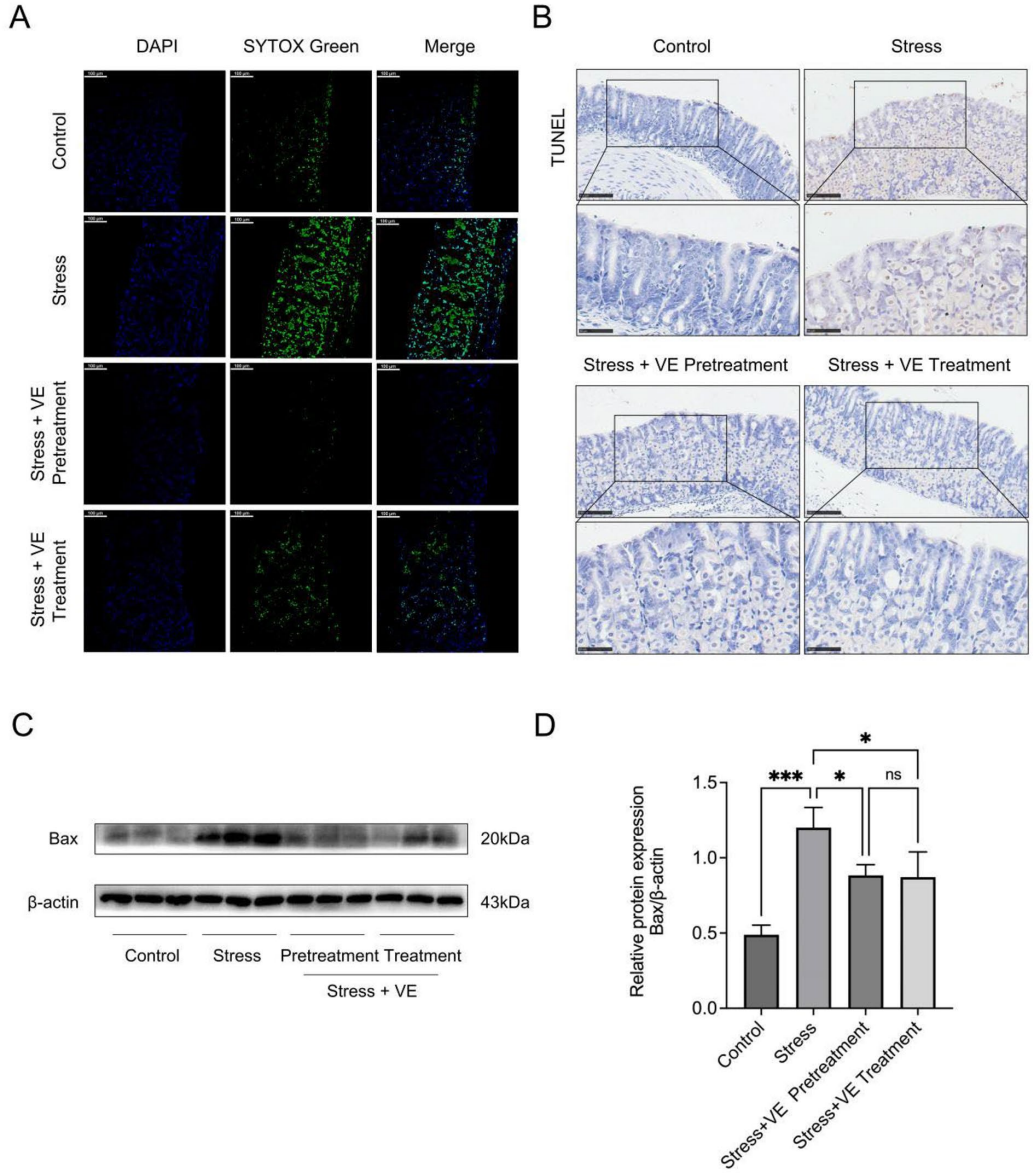
VE reduces apoptosis in stress‐induced gastritis. (A) Representative SYTOX green staining images in different groups are shown. Magnification: ×20, scale bar =100 μm. (B) Necrosis‐induced DNA fragmentation in gastric tissues was detected by TUNEL assay. Representative photographs in different groups were presented. Magnification: ×20, scale bar =100 μm; magnification: ×40, scale bar =50 μm. (C) Protein expression of Bax. (D) The quantification of western blot of Bax. All data are experssed as the mean ± SD. *p < 0.05, ***p < 0.001.

To further confirm whether VE gavage is able to delay stress‐induced gastritis progression, we conducted tests related to apoptosis in gastric tissue. So as to explore the integrity of cell membranes, SYTOX green fluorescent dye, a cationic dye capable of passing through the damaged membrane, was used to demonstrate the idea. As shown in Figure 4A, high‐intensity fluorescence signals could be observed in the group with simple stress, while weaker fluorescence signals were reflected in the control group and the groups with prophylactic and therapeutic administration. It was indicated that the number of cell membranes destroyed in the stomach tissue with VE pretreatment and treatment was significantly reduced or parallel to that of the control. As for the TUNEL assay, the results in Figure 4B showed that, under the same stimulation, the apoptosis of the two groups treated with drug prevention and treatment was notably reduced. As we can observe in Figure 4C,D. it was explored that the expression of pro‐apoptotic factor Bax in the stress group was increasing significantly (*p* < 0.001). Furthermore, the protein expression of Bax in the groups with VE gavage apparently reduced in accordance with expectation when compared to the stress group (*p* < 0.05). That said, there were no statistically significant differences between the pretreatment and treatment groups in terms of the protein expression of Bax (*p* > 0.05). From the above comprehensive analysis, it was demonstrated that VE reduces apoptosis and evidently alleviated the stress‐induced gastritis progression.

## 4 Discussion

In recent years, there has been widespread discussion among people about stress‐induced diseases, including stress‐induced gastritis [23, 24, 25, 26]. As described above, oxidative stress and inflammatory response are reportedly the major pathogenesis of stress‐induced gastritis. At present, VE holds a certain position in the prevention and treatment of inflammatory diseases due to its outstanding antioxidant and anti‐inflammatory effects that have been discovered [12, 27, 28]. Previous study has shown that VE has a sharp inhibitory effect on gastric mucosal damage in rats induced by water immersion‐restraint stress (WIRS) [29]. This study aims to explore the protective or therapeutic effects of VE on stress‐induced gastritis and its underlying mechanisms by constructing a mouse model of stress. It is suggested that VE is capable of apparently improving the gastric mucosal damage caused by stress by activating Nrf2/Hmox1/NQO1 and inhibiting NF‐κB signalling.

There has been verified that VE can reduce oxidative stress parameters and inflammatory markers [28]. It was also reported that VE resists oxidative stress by clearing free radicals, reducing singlet oxygen and superoxide [30, 31]. VE analogue has been revealed that it can alleviate oxidative stress by promoting the expression of Nrf2 and Hmox1, while defending the body against inflammation via inhibiting the activation of NF‐κB [32].

Related studies have noted that the Nrf2/Hmox1 pathway plays a crucial role in suppressing oxidative stress [33, 34]. Under the conditions of oxidative stress, oxidation of sulfhydryl groups on Keap1 leads to conformational changes, along with the release of Nrf2, which helps enhance the antioxidant stress response so that oxidative injury reduces [35, 36, 37]. Afterwards, genes with a similar inhibitory effect on oxidative stress were also activated, such as Hmox1 and NQO1, which serve as downstream molecules of Nrf2 [38]. Hmox1, the most important antioxidant factor, plays a vital role in regulating cellular redox balance [39]. Similarly, NOQ1 is able to avoid oxidative damage by removing superoxides [40]. We confirmed through immunohistochemical staining that VE increased the expression of Nrf2. Besides that, the results of q‐PCR demonstrated that VE upregulated the expression of Hmox1 and NQO1 mRNA. Consequently, it can be considered that the Nrf2/Hmox1/NQO1 pathway is most likely responsible for mediating the oxidative stress‐resistant properties of VE in stress‐induced gastritis. VE displayed significant antioxidant properties in stress‐induced gastritis via activating the Nrf2/Hmox1/NQO1 pathway.

As mentioned earlier, NF‐κB is a critical signalling pathway in the inflammatory response, and its activation plays a pivotal role in promoting the progression of inflammation [7, 8, 9, 10, 11]. The key to gastric mucosal damage lies in the inflammatory response [41]. Based on current research, the significant reduction of inflammatory markers in mice after VE pretreatment and treatment may be related to the powerful anti‐inflammatory properties of VE [12, 27]. Macrophages are particularly crucial in the onset and development of inflammatory diseases mediated by various factors [42]. In the early stages of inflammation, macrophages are capable of polarising into classic M1 macrophages, which promote the release of inflammatory factors such as IL‐1β [43, 44]. The result of western blot showed that the activation of the NF‐κB signal pathway was inhibited, as reflected by the decreased phosphorylation levels of p65. Consistent with our study, we proved that the infiltration of macrophages was substantially reduced and the expression level of the pro‐inflammatory mediator IL‐1β was downregulated in stress‐induced gastritis models treated with VE prevention and treatment. Upon histopathological findings, compared with the simple stress group, VE dramatically reduced inflammatory cell infiltration and oedema degree, which further confirmed the improvement of gastric mucosal inflammation.

In this study, we also compared the effects of VE as both a protective agent and a treatment for stress‐induced gastritis. Our results showed no statistically significant differences between the pretreatment and treatment groups for key indicators, which indicates that while VE demonstrated protective effects, its impact as a treatment did not differ significantly from its preventive role under the conditions tested. However, it does not imply that there is no difference between preventive and therapeutic administration, but rather reflects the specific experimental conditions. This may be related to factors such as the timing of administration and requires further investigation in future studies.

There is a growing evidence proving that Nrf2/Hmox1 is related to NF‐κB. Based on previous research, the activation of Nrf2/Hmox1 and the inhibition of NF‐κB form mutual feedback [45]. Normally, the upregulation of Hmox1 caused by the activation of Nrf2 can restrict NF‐κB activity by inhibiting IκB degradation, so the expression of inflammatory factors decreased [46, 47]. On the contrary, a report suggested that NF‐κB competitively leads to Nrf2 inactivation [48]. However, further research and validation are needed to investigate the interaction between the two signalling pathways caused by vitamin E in diseases.

The present study has evaluated a beneficial role of VE in the prevention and management of stress‐induced gastritis in mice. According to macroscopic examination, both VE pretreatment and treatment have obviously improved the haemorrhagic injury of gastric mucosa. In addition, it was also supported by histopathological findings. These results are consistent with a previously published study [29]. The related investigation of the detection of pro‐apoptotic factor, TUNEL assay and SYTOX green staining was used to further confirm this conclusion.

## 5 Conclusion

**FIGURE 5:**
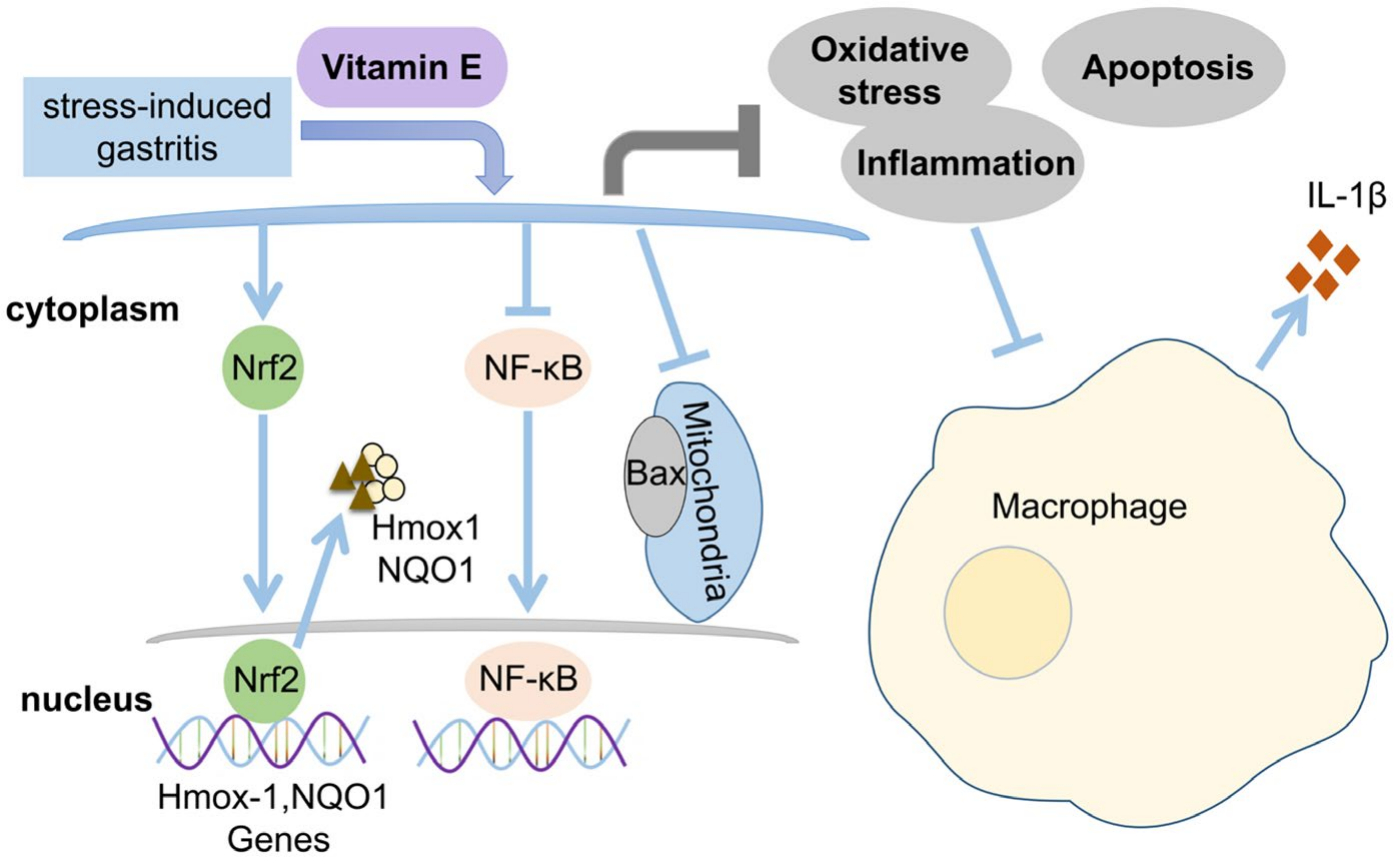
The protective effect and underlying mechanism of VE in stress‐induced gastritis.

In summary, the prevention and therapy of VE have significant protective effects on stress‐induced gastritis via promoting Nrf2/Hmox1/NQO1 and inhibiting NF‐κB signalling pathways. In addition, VE reduces apoptosis in stress‐induced gastritis. It has extremely powerful antioxidant and anti‐inflammatory effects on the progression of the disease (Figure 5). Based on these results, we suggest that VE may contribute to the prevention and treatment of stress gastritis in the future.

## Author Contributions

**Xiaolin Xie:** conceptualization (equal), data curation (equal), formal analysis (equal), investigation (equal), methodology (equal), project administration (equal), software (equal), writing – original draft (equal), writing – review and editing (equal). **Si Zhao:** conceptualization (equal), data curation (equal), formal analysis (equal), methodology (equal), writing – review and editing (equal). **Rui Fang:** conceptualization, data curation, formal analysis, investigation, methodology, writing – review and editing (equal). **Huan Chen:** data curation, formal analysis, investigation, software, writing – original draft, writing – review and editing (equal). **Han Zhang:** data curation, formal analysis, software, writing – review and editing (equal). **Xue Wang:** formal analysis, investigation, writing – review and editing (equal). **Jun Gao:** writing – original draft (equal), writing – review and editing (equal). **Yan Liu:** writing – original draft (equal), writing – review and editing (equal). **Zihao Cai:** writing – original draft (equal), writing – review and editing (equal). **Ming Zhang:** project administration, supervision (equal), writing – review and editing (equal). **Bing Xu:** conceptualization, methodology, project administration, supervision, writing – review and editing. **Yuzheng Zhuge:** funding acquisition (lead), supervision (equal), validation (equal), writing – review and editing (equal).

## Conflicts of Interest

The authors declare no conflicts of interest.

## Data Availability

The data that support the findings of this study are available within the manuscript.
